# Cyclohexylmethyl Flavonoids Suppress Propagation of Breast Cancer Stem Cells via Downregulation of NANOG

**DOI:** 10.1155/2013/170261

**Published:** 2013-04-04

**Authors:** Wen-Ying Liao, Chih-Chuang Liaw, Yuan-Chao Huang, Hsin-Ying Han, Hung-Wei Hsu, Shiaw-Min Hwang, Sheng-Chu Kuo, Chia-Ning Shen

**Affiliations:** ^1^Stem Cell Program, Genomics Research Center, Academia Sinica, Nangang, Taipei 115, Taiwan; ^2^Graduate Institute of Pharmaceutical Chemistry, China Medical University, Taichung 402, Taiwan; ^3^Department of Marine Biotechnology and Resources, National Sun Yat-sen University, Kaohsiung 804, Taiwan; ^4^Asia-Pacific Ocean Research Center, National Sun Yat-sen University, Kaohsiung 804, Taiwan; ^5^Department of Biotechnology and Laboratory Science in Medicine, National Yang-Ming University, Taipei 112, Taiwan; ^6^Bioresource Collection and Research Center, Food Industry Research and Development Institute, Hsinchu 300, Taiwan; ^7^The Ph.D. Program for Cancer Biology and Drug Discovery, China Medical University, Taichung 402, Taiwan; ^8^Graduate Institute of Clinical Medicine, Taipei Medical University, Sinyi District, Taipei 110, Taiwan

## Abstract

Breast cancer stem cells (CSCs) are highly tumorigenic and possess the capacity to self-renew. Recent studies indicated that pluripotent gene *NANOG* involves in regulating self-renewal of breast CSCs, and expression of NANOG is correlated with aggressiveness of poorly differentiated breast cancer. We initially confirmed that breast cancer MCF-7 cells expressed NANOG, and overexpression of NANOG enhanced the tumorigenicity of MCF-7 cells and promoted the self-renewal expansion of CD24^−/low^CD44^+^ CSC subpopulation. In contrast, knockdown of NANOG significantly affected the growth of breast CSCs. Utilizing flow cytometry, we identified five cyclohexylmethyl flavonoids that can inhibit propagation of NANOG-positive cells in both breast cancer MCF-7 and MDA-MB231 cells. Among these flavonoids, ugonins J and K were found to be able to induce apoptosis in non-CSC populations and to reduce self-renewal growth of CD24^−/low^CD44^+^ CSC population. Treatment with ugonin J significantly reduced the tumorigenicity of MCF-7 cells and efficiently suppressed formation of mammospheres. This suppression was possibly due to p53 activation and NANOG reduction as either addition of p53 inhibitor or overexpression of NANOG can counteract the suppressive effect of ugonin J. We therefore conclude that cyclohexylmethyl flavonoids can possibly be utilized to suppress the propagation of breast CSCs via reduction of NANOG.

## 1. Introduction

Breast cancer is a leading cause of cancer death among women, as cancer recurrence and metastasis occur frequently in breast cancer patients [1, 2]. Accumulating evidence indicates that CD24^-/low^CD44^+^ breast cancer cells, also referred to as “tumorigenic breast cancer cells” [3, 4], “breast cancer stem cells (CSCs)” [5], and “stem-like breast cancer cells” [6], possess stem cell characteristics, display resistance to conventional therapies, and have high tumor-initiating and metastatic ability [3, 4, 7–9]. Therefore, the presence of breast CSCs has been suggested to be the underlying cause of breast cancer recurrence and metastasis [2, 8, 9]. In order to improve breast cancer therapeutics, efforts are now being directed towards identifying strategies that target breast CSCs [2, 9].

Accumulating evidence supports that self-renewal regulators of normal stem cells may govern clinical behavior of human cancer [10, 11]. For example, embryonic stem cell (ESC) signature is associated with poor clinical outcome in patient of breast cancer patients [12]. Among the regulatory genes involved in pluripotent maintenance of ESCs, NANOG was found to express a NANOGP8 retrogene locus in a wide variety of somatic and cancer cells [13–15]. Recent work has shown that NANOG was functionally involved in human tumor development and in regulating cancer stemness [15, 16]. Knockdown of NANOG significantly reduced the tumorigenic potentials of various cancer cells including breast cancer [17]. NANOG has also been identified in breast cancer cells and was found to mediate multidrug resistance via activation of STAT3 signaling [18] suggesting that NANOG is a potential target for breast cancer therapeutics.

Herbal medicine has been proposed for utilizing a complementary approach for control of breast cancer recurrence and metastasis [19, 20]. However, whether the activity of breast CSCs can be suppressed by treatment of herbal medicine has never been addressed. In Chinese traditional medicine, the roots of the fern *Helminthostachys zeylanica *(L.) Hook. (Ophioglossaceae), known as “Ding-Di-U-Gon”, is used as antipyretic and antiphlogistic agent to treat inflammatory diseases, various hepatic disorders, and possibly malignancy in pancreas [21–23]. The rhizome of this medicinal fern is also named as “tunjuk langit” in India which has been used as a folk medicine to treat pulmonary disease and even to cure impotency by the tribal people [24]. In Malaysia, the rhizome is used as an antidiarrheal agent and chewed with areca for whooping cough relief [25]. However, efforts to evaluate the efficacy of such treatment on CSCs and to identify responsible principles of its effect on cancer were scarce. 

In the present study, a group of natural cyclohexylmethyl flavonoids isolated from the rhizomes of *H. zeylanica* had been examined. Utilizing flow cytometry, we identified five members of natural cyclohexylmethyl flavonoids that can inhibit expansion of NANOG^+^ cells. Among these cyclohexylmethyl flavonoids, ugonins J and K, which were the main components of the ethyl acetate-soluble extract of the rhizomes of *H. zeylanica*, were able to suppress propagation of CD24^-/low^CD44^+^ breast cancer stem cells both *in vitro* and *in vivo*.

## 2. Materials and Methods 

### 2.1. Cell Culture

Both human breast cancer cell lines MCF-7 and MDA-MB231 were obtained from Bioresource Collection and Research Center (Hsin-Chu, Taiwan) and maintained in either *α*-Minimum Essential Medium (*α*-MEM) or L-15 medium (Invitrogen) supplemented with 2 mM L-glutamine (Sigma), 1.5 g/L sodium bicarbonate, 0.1 mM nonessential amino acids (Invitrogen), 1.0 mM sodium pyruvate (Invitrogen), and 10% fetal bovine serum (FBS) (Invitrogen). Human foreskin fibroblast HFF-1 cells were imported from ATCC and were maintained in ATCC-formulated Dulbecco's modified Eagle's medium supplemented with 15% FBS (Invitrogen).

### 2.2. Chemicals

Doxorubicin (Dox) was obtained from Sigma. Ugonins (J-S) were isolated and purified from the rhizomes of *Helminthostachys zeylanica* [21]. All of the ugonins used in the experiments were repurified by reversed-phase HPLC to ensure the purity >99%.

### 2.3. Formation of Mammospheres

MCF-7 cells (1 × 10^4^ cells) were grown in suspension culture in serum-free Dulbecco's Modified Eagle Medium (DMEM) supplemented with 2 mM-L-glutamine, 0.1 mM nonessential amino acids, 20 ng/mL human epidermal growth factor (R&D), 20 ng/mL basic fibroblast growth factor (Millipore), 4 *μ*g/mL heparin, and 5 *μ*g/mL insulin (Sigma) and 1x B27 supplement (Invitrogen).

### 2.4. Flow Cytometric Analysis

Cells were trypsinized and washed three times with PBS before resuspension in Hanks' Balanced Salt Solution (HBSS; Invitrogen) containing 2% FBS and 10 mM HEPES (Invitrogen). The cell density was adjusted to 10^6^/100 *μ*L in staining buffer before being stained with antibodies FITC-conjugated anti-CD24 (BD Biosciences) and APC-conjugated anti-CD44 (BD Biosciences) for 30 minutes. In some experiments, MCF-7 cells were stained with anti-NANOG antibodies (Cell Signaling) followed by staining with FITC-conjugated goat anti-rabbit IgG (BD Biosciences). Stained cells were analyzed utilizing FACSCalibur flow cytometry (BD Biosciences) after the addition of propidium iodide (2 *μ*g/mL) to exclude dead cells.

### 2.5. Immunofluorescent Staining

MCF-7 cells (5 × 10^4^ cell/well) were seeded in the 24-well plate and cultured overnight. After cells were treated with different compounds for different time course, cells were fixed by 4% PFA (Sigma) for 30 minutes at room temperature and permeabilized at room temperature in 0.1% Triton X-100 for 30 minutes. After blocking with 2% Roche blocking reagent, the cells were incubated with primary antibody overnight at 4°C and with secondary antibody for 2 hours at room temperature. The primary antibodies were used at the following dilutions: rabbit anti-NANOG 1 : 100 (Cosmo Bio USA, Inc) and rabbit antiphospho-p53^S15^ 1 : 400 (Cell Signaling). Cells were counter-stained with Hoechst dye (Sigma) to visualize the cell nuclei. Images of the immunostaining were obtained using a fluorescence microscopy (Leica Microsystems Inc).

### 2.6. Establishment of NANOG-Overexpressing and p53-Overexpressing Cells

The lentiviral construct-pSin-EF2-NANOG-Pur was obtained from Addgene (plasmid 16578) [26]. In order to produce NANOG lentivirus, the day prior to transfection, 293T cells were seeded at 2.4 × 10^6^ cells per 10-cm dish. Each 10-cm dish was transfected with 7.5 *μ*g pSin-EF2-Nanog-Pur 6.75 *μ*g pCMV-Δ8.91 packaging plasmid, and 0.75 *μ*g pMD.G envelope plasmid using Genejuice transfection reagent (Novagen). Virus-containing supernatant was collected and filtered through 0.45 *μ*m pore filters and stored at 4°C. Virus was further concentrated by ultracentrifugation for 2.5 hours at 26000 rpm in a Beckman SW 28.1 rotor (Beckman Coulter), and the resulting virus pellet was resuspended in PBS (pH 7.4) containing 1% BSA at 4°C overnight before being aliquoted and stored at −80°C. MCF-7 cells were first infected with NANOG lentivirus and then NANOG-overexpressing cells were selected in *α*-MEM containing 1 *μ*g/mL puromycin. The GFP-p53 plasmid was obtained from Addgene (plasmid 12091) [27]. MCF-7 cells (5 × 10^4^ cell/well in 24-well plate) were seeded on coverslips and transfected with 0.25 *μ*g of GFP-p53 plasmid using GeneJuice reagent (Merck Millipore). 

### 2.7. Establishment of NANOG-Knockdown Cells

The lentiviral shNANOG construct (TRCN0000004884) was obtained from the National RNAi Core Facility (Institute of Molecular Biology/Genomic Research Center, Academia Sinica), and the lentivirus was generated as described in the previous section. MCF-7 cells were infected with shNANOG lentivirus, and then NANOG-knockdown cells were selected in *α*-MEM containing 1 *μ*g/mL puromycin.

### 2.8. Western Blotting Analysis

Whole-cell extracts were prepared using RIPA buffer containing 150 mM NaCl, 50 mM Tris HCl (pH 8), 1% NP-40, 0.5% sodium deoxycholate, 0.1% SDS, and protease inhibitors and phosphatase inhibitors cocktails (Sigma). Whole-cell extracts of MCF-7 cells were separated by 10% SDS-PAGE and subsequently transferred to PVDF membrane (Millipore). Samples were incubated in blocking buffer (0.1% Tween 20, 5% nonfat milk powder in TBS) for 1 hour at room temperature. Afterwards, the membrane was incubated with primary antibody in blocking buffer overnight at 4°C before being washed twice with TBST (0.1% Tween in TBS) and incubated with the appropriate secondary antibody in blocking buffer for 1 hour at room temperature. The blot was developed using ECL western blotting substrate (Millipore) and analyzed using the luminescent image analyzer, LAS-4000mini (Fujifilm). The primary antibodies were used at the following dilutions: rabbit anti-NANOG, 1 : 1000 (Cell Signaling); rabbit anti-p53, 1 : 1000 (Cell Signaling); rabbit anti-p53-Ser15p, 1 : 1000 (Cell Signaling); rabbit anti-p53-Ser392p, 1 : 1000 (Cell Signaling); rat anti-ABCG2, 1 : 100 (Abcam); rabbit anti-Stat3, 1 : 2000 (Cell Signaling); rabbit antiphospho-Stat3^Y705^, 1 : 1000 (Cell Signaling); rabbit antiphospho-Stat3^S727^, 1 : 1000 (Cell Signaling); rabbit anticleaved PARP, 1 : 1000 (Cell Signaling); rabbit anticleaved Caspase9, 1 : 1000 (Cell Signaling); and mouse anti-*β*-actin, 1 : 10000 (Sigma). The secondary antibodies used were anti-rabbit HRP (1 : 1000, Santa Cruz) or anti-mouse HRP (1 : 1000, Santa Cruz). 

### 2.9. Analysis of the Promoter and p53-Binding Site of NANOG and NANOGP8

To analyze the elements upstream of NANOG and NANOGP8, the 5-kb upstream sequences of the translation start sites of NANOG and NANOGP8 were retrieved from the human RefSeq files (NC_000012 and NC_000015, resp.). The *p53MH* program (PMID: 12077306) was employed to detect possible P53-binding site within the 5-kb sequence. The top 100 possible p53-binding sites were extracted. For the identification of the most likely binding site, the threshold of the percentage of maximum possible score was set as 80%. The prediction of the promoter region was carried out with *CoreBoost_HM* (PMID: 18997002). The score of 0.7 was set as a cutoff value for the plausible promoter region.

### 2.10. Establishment of Orthotropic Tumor Xenografts in SCID Mice

All animal experiments were approved by the Academia Sinica Institutional Animal Care and Utilization Committee. Four-week-old female SCID mice purchased from BioLASCO were used to carry out MCF-7 xenograft experiments. For tumorigenicity assay, eighteen mice were divided into three groups (6 mice/group) and were injected in the mammary fat pad with Control, NANOG-overexpressing, or NANOG-knockdown MCF-7 cells (1 × 10^6^ cells/60 *μ*L). To determine if ugonin J can suppress tumor growth, eighteen mice were divided into three groups (6 mice/group) and were injected in the mammary fat pad with MCF-7 cells (2 × 10^5^ cells/60 *μ*L). When the tumor volume reached 50 mm^3^ (set as Day 0), the tumor-bearing mice were then administered a weekly dose of doxorubicin (12 mg/kg, dissolved in 100 *μ*L of DMSO) or ugonin J (50 mg/kg, dissolved in 100 *μ*L of DMSO) interperitoneally for a total of 4 doses. Body weight of mice and tumor size were measured weekly. 

### 2.11. Histology and Immunohistochemistry

Tumor tissues were fixed overnight at room temperature with 3.5% formaldehyde solution containing 68.6% EtOH and 4.8% acetic acid (FAA fixative) prior to being processed and embedded in paraffin. 4 *μ*m thick sections were cut and mounted on Superfrost plus slides (Thermo Scientific). For immunohistochemical staining, sections were subjected to antigen retrieval in Citric-acid based buffer (Vector Laboratories) at 95°C for 20 minutes. The sections were then permeabilized with 0.1% (v/v) Triton X-100 in PBS for 30 min and incubated in 2% blocking buffer (Roche) before being incubated sequentially with primary, HRP-conjugated secondary antibodies. Super Sensitive Polymer HRP IHC Detection System (Biogenex Laboratories) was used to visualize the positive cells. Sections were counterstained with hematoxylin and mounted with Entellan Neu (Merck). The primary antibodies were used at the following dilutions: rabbit anti-Nanog 1 : 150 (Cosmo Bio), mouse anti-MUC 1 1 : 100 (Abcam), and mouse anti-HCAM/CD44 1 : 100 (SantaCruz). 

### 2.12. Invasion Assay

1 × 10^4^ of MCF-7 cells suspended in serum-free medium with or without ugonins J or K was seeded into the top chamber of the matrigel-coated insert (Millicell, 24-well plate, 8 *μ*m, Millipore) in 100 *μ*L serum-free medium. In the lower chamber, the well was filled with serum-containing medium which was used as a chemoattractant. After 24-hour incubation, cells that did not invade through the pores were removed by a cotton swab. Cells on the lower surface of the membrane were fixed with methanol and stained with Giemsa solution (Merck). The number of invasive cells/each well was counted under a light microscope. Data are representative of three independent experiments. ***P < 0.001, **P < 0.01 versus compared control.

### 2.13. Statistical Analysis

Experiments were repeated at least three times with consistent results. Statistical differences between groups were determined by unpaired Student's *t* test. The statistical significance was set at *P < 0.05, **P < 0.01, ***P < 0.001. FACS data were analyzed by FlowJo software (Ashland, OR, USA). The statistical analysis for fluorescent staining used MetaMorph imaging analytical software (Molecular Devices).

## 3. Results 

### 3.1. A Critical Role of NANOG in Modulating Proliferation and Tumorigenicity of Breast Cancer Cells

**Figure 1:**
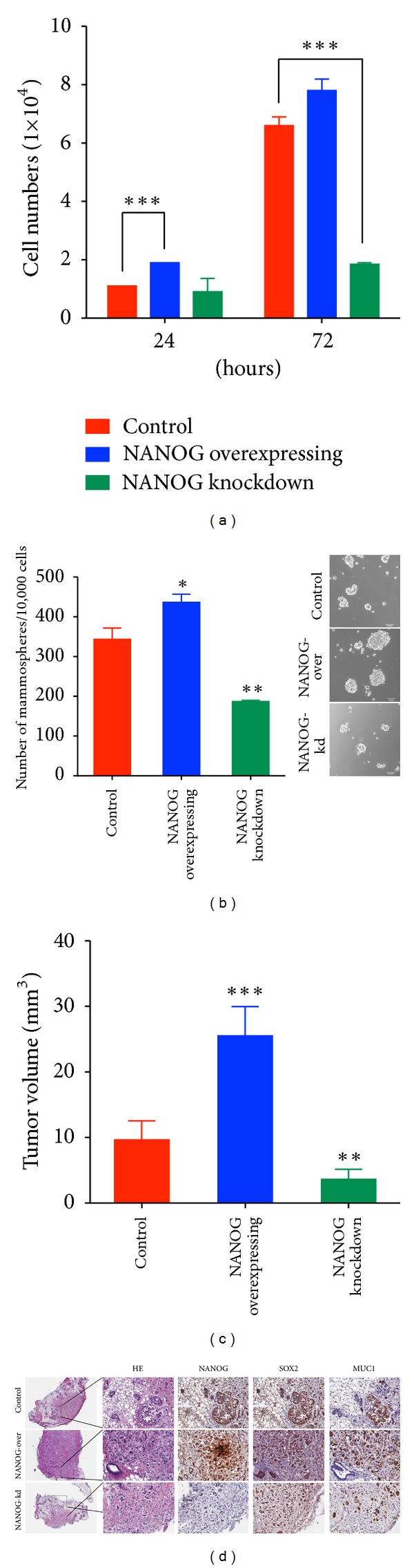
NANOG expression plays an important role in cell proliferation and tumorigenesis. (a) Total cell number of MCF-7, NANOG-overexpressing MCF-7, and NANOG-knockdown MCF-7 cells (0.9 × 10^5^ cells in 12-well plates) were counted after 24, 72 hours of culture (*n* = 3). (b) Mammosphere formation in sphere-forming medium for 28 days. Total mammospheres were counted under a microscope at days 28. Mean of three independent experiments ± SEM. **P < 0.01, *P < 0.05 versus control mammospheres. (c) Eighteen SCID mice were divided into three groups (6 mice/group). The MCF-7 orthotopic tumors in SCID mice were formed with vector control, NANOG-overexpressing, and NANOG-knockdown MCF-7 cells (1 × 10^6^). The tumor volumes of SCID mice were measured weekly. The average tumor volume of MCF-7 tumors was removed from SCID after 4 weeks. ***P < 0.001, **P < 0.01 versus vector control (d) NANOG overexpression enhanced expression of the cancer stem cell marker SOX2 and MUC1 in tumor xenografts. Hematoxylin-Eosin stain and Immunohistochemical detection (x200) for NANOG, SOX2 and MUC1 on vector control, NANOG-overexpressing and NANOG-knockdown tumor xenografts.

We initially investigated whether expression of NANOG plays an important role in breast cancer growth. To address this question, we generated NANOG-overexpressing and NANOG-knockdown MCF-7 cell lines. As shown in Figure 1(a), RNA interference-mediated NANOG knockdown reduced breast cancer. And overexpression of NANOG slightly increased the overall growth rate. To further determine if NANOG is the key component modulating self-renewal capability and tumorigenicity of the tumorigenic breast cancer cells, we carried out the mammosphere-forming assay and orthotropic tumor xenografts experiments in female SCID mice. As shown in Figures 1(b) and 1(c), NANOG-overexpressing cells formed 20% more of mammospheres and generated twofold larger tumor xengrafts than control MCF-7 cells. In contrast, knockdown of NANOG not only significantly reduced the ability to form mammospheres, but also dramatically reduced the tumorigenicity of MCF-7 cells. Immunohistochemical analysis of tumor xengrafts (Figure 1(d)) further confirmed that NANOG overexpression enhanced tumor development and increased expressions of cancer stemness protein-SOX2 and MUC1 in tumor xengrafts. Oppositely, NANOG-knockdown cells generated tiny tumor nodules with lower levels of SOX2 and MUC1. 

**Figure 2:**
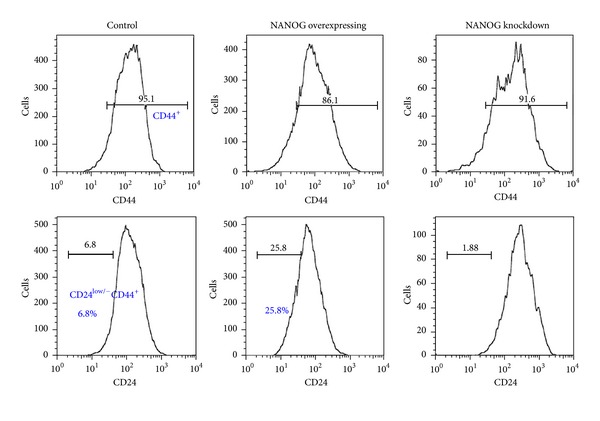
NANOG overexpression enhanced propagation of cancer stem cells. Control, NANOG-overexpressing and NANOG-knockdown MCF-7 cells were double-stained with anti-CD24 and anti-CD44 antibodies followed by FACS analysis (*n* > 3). CD44^+^ cell population (top panel) and CD24^−^/low in CD44^+^ cell population (bottom panel) were analyzed.

Since NANOG knockdown suppressed mammosphere formation and reduced levels of SOX2 and MUC1 in tumor xengrafts, we next tried to determine if propagation of CD24^−/low^CD44^+^ breast CSC subpopulation in MCF-7 cells is also regulated by NANOG [3, 4]. As shown in Figure 2, we found that overexpression of NANOG increased the proportion of CD24^−/low^CD44^+^ CSC subpopulation in MCF-7 cells from 6.8% to 25.8%. In contrast, NANOG knockdown reduced the proportion of CD24^−/low^CD44^+^ CSC subpopulation in MCF-7 cells from 6.8% to 1.88%. These data indicated that NANOG played an important role in modulating self-renewal and tumorigenicity of breast CSC subpopulations. 

### 3.2. Identification of Bioactive Cyclohexylmethyl Flavonoids Targeting NANOG^+^ Breast Cancer Cells

**Figure 3:**
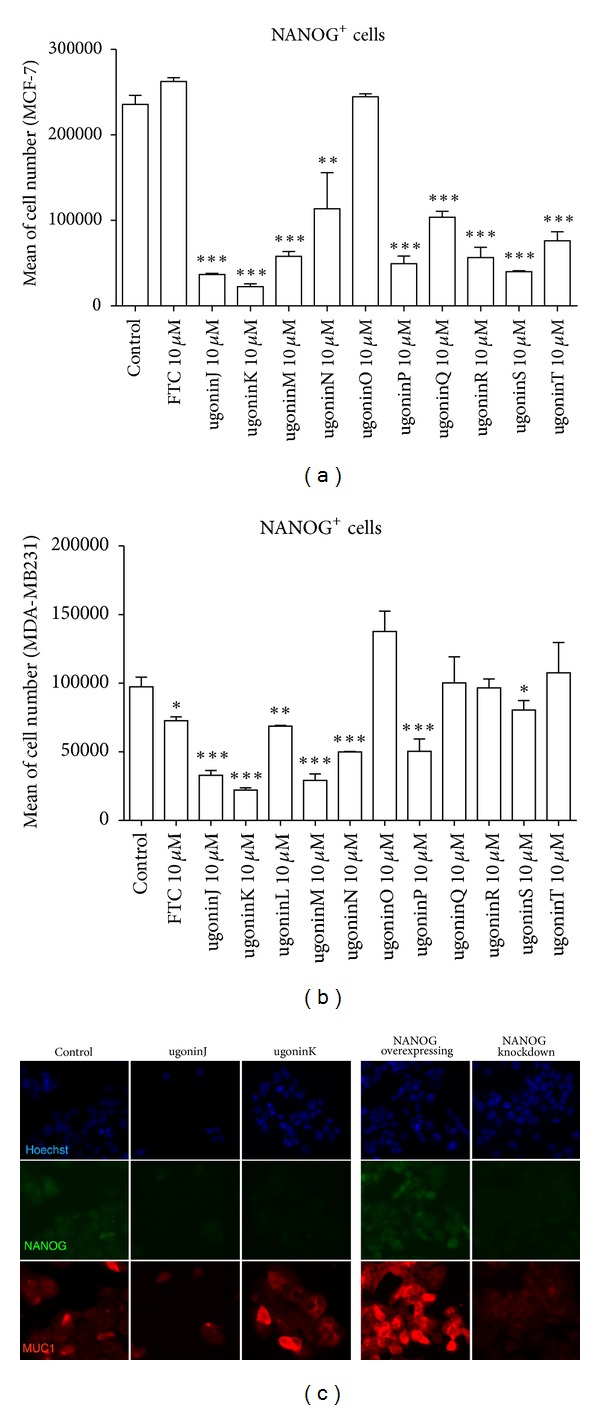
Natural product screening to reduce NANOG^+^ subpopulation of MCF-7 cells. ((a) and (b)) Screening for natural products by reducing NANOG^+^ population assay. MCF-7 cells and MDA-MB231 cells (9 × 10^4^ cells in 12-well plates) were treated with natural products for 72 hours before NANOG levels were measured. Total NANOG^+^ cells were calculated and data were shown as mean ± SEM from 3 independent experiments. ***P < 0.001 or **P < 0.01 versus control cells. (c) Immunofluorescent staining of NANOG (green) and MUC1 (red) on control, treated with ugonins (J, K), NANOG-overexpressing, and NANOG-knockdown MCF-7 cells.

We have explored that NANOG possibly played a critical role in modulating self-renewal expansion and tumorigenicity of breast CSCs. We therefore then assume identification of bioactive natural components from herb medicine that can suppress that NANOG would be beneficial for developing a complementary approach for control of breast CSC-driven recurrence and metastasis. Since the dietary flavonoids were reported to possess the ability to suppress the prostate CSCs via inhibiting NANOG [28], a group of natural cyclohexylmethyl flavonoids isolated from the rhizomes of *H. zeylanica* that had been examined. Initially, an MTT colorimetric assay was used to determine cytotoxicity of cyclohexylmethyl flavonoids to two breast cancer cell lines (MCF-7 and MDA-MB231) (Table 1). Among these flavonoids, ugonins J and K were found to display cytotoxicity (IC_50_ < 25 *μ*M) to breast cancer cells. In contrast, these two ugonins were less cytotoxic to normal foreskin fibroblasts (HFF). Utilizing flow cytometry, we identified five members of natural cyclohexylmethyl flavonoids that inhibited expansion of NANOG^+^ population in both MCF-7 and MDA-MB 231 cells (Figures 3(a) and 3(b)). Among these natural cyclohexylmethyl flavonoids, based on using immune-fluorescent staining, we validated that either treatment of ugonins J or K, both compounds were the main component of the ethyl acetate-soluble extract of the rhizomes of *H. zeylanica*, significantly reduced the expression level of NANOG and MUC1 in MCF-7 cells (Figure 3(c)). 

### 3.3. Downregulation of NANOG Mediates the Suppressive Effect of Ugonin J on Propagation of Breast Cancer Stem Cells

**Figure 4:**
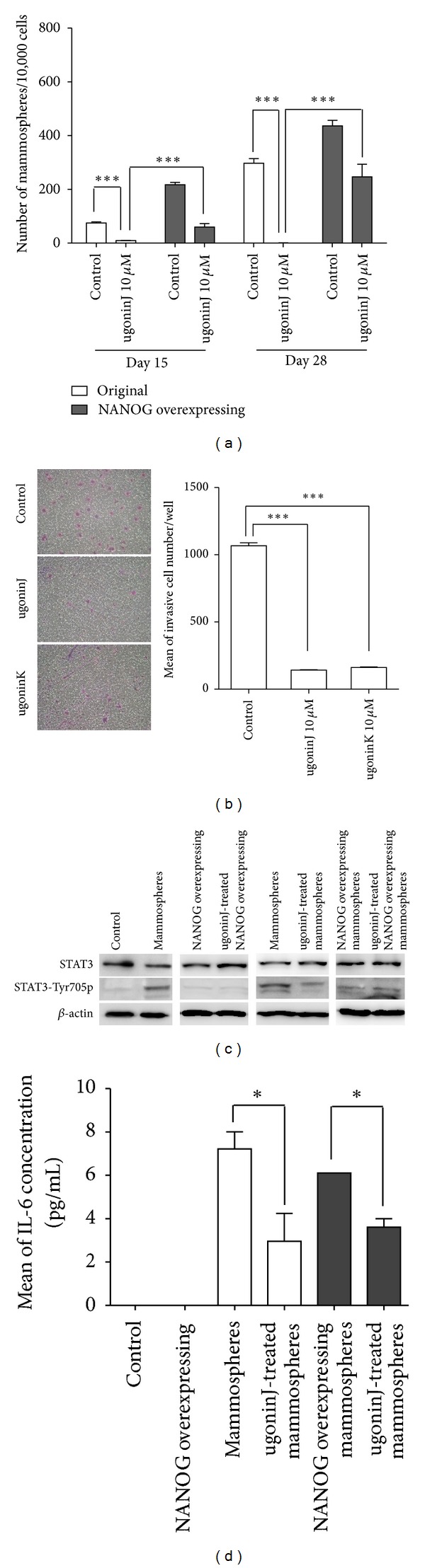
NANOG overexpression counteracts the suppressive effect of ugonin J on propagation of breast cancer stem cells. (a) Control and NANOG-overexpressing MCF-7 cells (1 × 10^4^ cells in 24-well plates) were treated with ugonin J for 3 days prior to mammosphere formation in mammosphere forming medium for 28 days. Total mammospheres were counted under a microscope at days 15 and 28. Mean of three independent experiments ± SEM. ***P < 0.001 versus control mammospheres. (b) Invasion assay of MCF-7 was performed on matrigel with or without ugonins J or K treatment. Data was shown as mean ± SEM from three independent experiments. ***P < 0.001, **P < 0.01 versus control. (c) Control, NANOG-overexpressing cells (4 × 10^5^ cells in 6-well plates), mammospheres, and NANOG-overexpressing mammospheres (1000 spheres in 6-well plates) were treated with ugonin J for 24 hours before protein extraction. Western blot probed for STAT3 and phospho-STAT3 Tyr705. Equal amounts of protein were used (40 *μ*g per lane). (d) Mammospheres were formed for 15 days from control and NANOG-overexpressing mammospheres (20 spheres in 96 plates) before treatment with or without 10 *μ*M ugonin J for 24 hours. Medium was collected and analyzed by ELISA to determine the production of IL-6 (*n* = 3). Data was shown as mean ± SEM. *P < 0.05 versus control.

The ability of formation of mammospheres is known as one of self-renewal characteristics of breast CSCs; we then determined if treatment of ugonin J can suppress mammosphere-forming ability. In comparing with NANOG overexpression increased mammosphere formation, pretreatment with ugonin J completely inhibited formation of mammospheres in control MCF-7 cells (Figure 4(a)). In contrast, NANOG overexpression partially counteracted the suppressive effect of ugonin J on mammosphere formation. We then determined if ugonin J can reduce the malignant features of MCF-7 cells including invasion ability, and IL-6 secretion led to STAT3 phosphorylation in mammospheres (Figure 4(b)) [29]. Treatment with ugonin J or K for 24 hours significantly suppressed invasion ability of MCF-7 cells. Moreover, treatment of 28-day mammospheres with ugonin J for 24 hours significantly reduced IL-6 secretion and STAT3 phosphorylation in both control mammospheres and NANOG-overexpressing mammospheres (Figures 4(c) and 4(d)). These results suggest that ugonin J-mediated downregulation of NANOG may be a key event affecting the propagation of breast CSCs. 

### 3.4. P53-Dependent Pathway Mediates Downregulation of NANOG by Ugonin J Treatment

**Figure 5:**
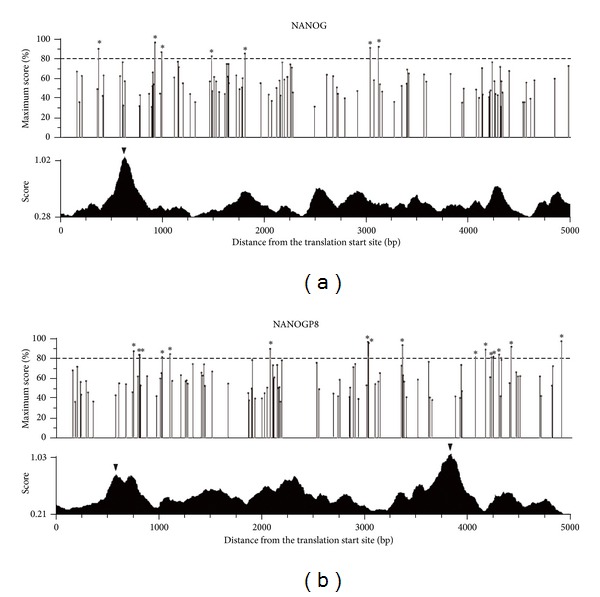
P53-binding site existed in the regulatory region of *NANOG *and *NANOGP8.* The upper panel presents the detection of the p53-binding site in the 5-kb upstream sequence of the translation start site of a gene. The percentage of maximum possible score stands for the possibility of being a p53-binding site. The cutoff value was set as 80% to unveil the p53-binding site candidates. The most likely p53-binding site is indicated by asterisk. The lower panel exhibits the prediction of the promoter region. The triangle indicates the possible promoter region with the score of more than 0.7. *P < 0.05 or **P < 0.01.

NANOG is a pluripotent regulator of embryonic stem cells, and previous studies have shown that p53 binds to the promoter of *NANOG* and suppresses *NANOG* expression after DNA damage [30, 31]. However, it has recently been shown that there are 11* NANOG* pseudogenes [32]. *NANOGP8* has been recognized as a retrogene and was recently found to be expressed in various cancer tissues and several cancer cell lines including breast cancer MCF-7 cells. We therefore determined if *NANOGP8* can also be regulated by p53. The *p53MH* program was employed to detect possible P53-binding site within the 5-kb sequence in the *NANOG* and *NANOGP8* promoter regions. As shown in Figure 5, both *NANOG* and *NANOGP8* promoter regions contained several potential binding site for p53. 

**Figure 6:**
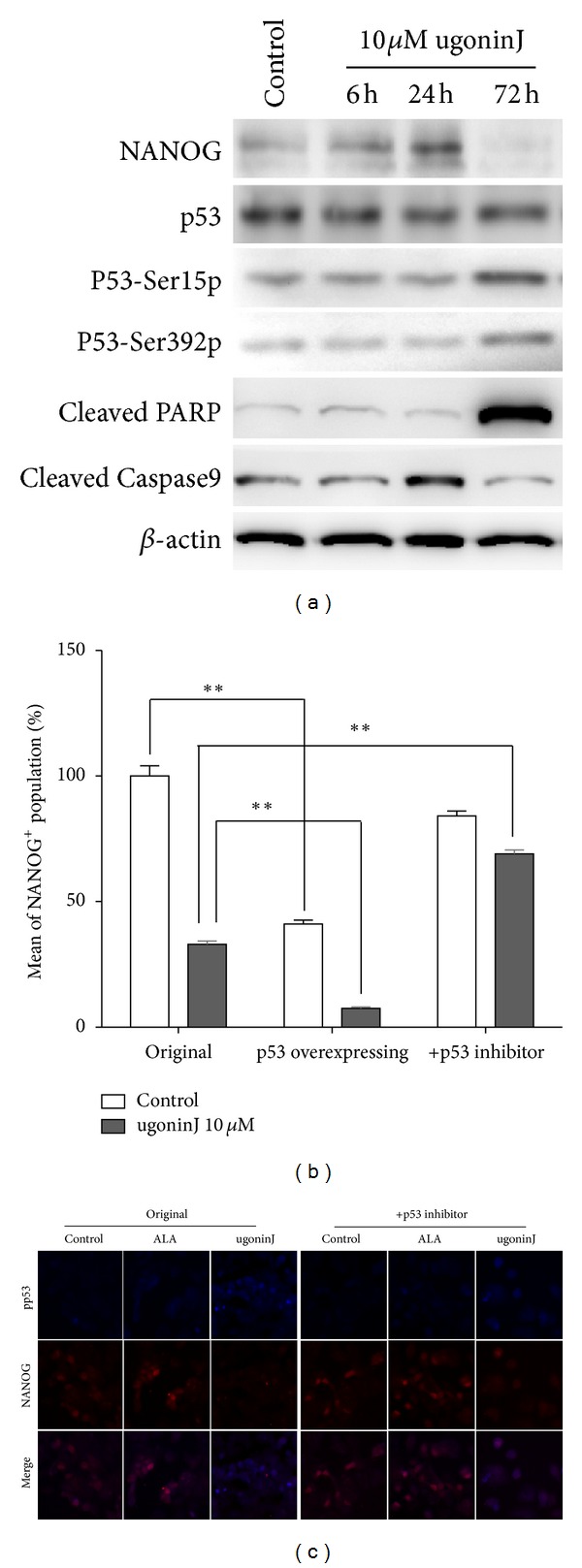
P53-dependent pathway mediates the downregulation of NANOG by ugonin J treatment in MCF-7 cells. (a) MCF-7 cells were treated with 10 *μ*M ugonin J for 6, 24, and 72 hours before protein extraction. Western blot probed for anti-ABCG2, NANOG, p53, phospho-p53 Ser15 and 392, and cleaved PARP and caspase 9 antibodies. Equal amounts of protein were used (40 *µ*g per lane). (b) Relative percentage of NANOG^+^ population in MCF-7, p53-overexpressing MCF-7, and pifithrin-*α* (p53 inhibitor)-treated MCF-7 (0.9 × 10^5^ cells in 12-well plates) were treated with 10 *μ*M ugonin J and counted after 48 hours of culture (*n* = 3). **P < 0.01 versus J-treated control. (c) Pifithrin-*α* treatment rescued the reductive effect of Nanog. MCF-7 cells were treated with ALA and ugonin J for 72 hours. Nanog (red) and phospho-p53^ser15^ (blue) expression was analyzed by immunofluorescent staining.

To further determine if p53 pathway can be activated by ugonin J treatment. Time-course experiments were performed and showed that treatment of ugonin J (Figure 6(a)) did in fact increase phosphorylation of p53 at ser15 and ser392 and also activated the apoptotic pathway, as evidenced by cleaved forms of Poly (ADP-ribose) polymerase (PARP) and caspase 9 in western blot analysis. In order to determine whether the downregulation of NANOG in MCF-7 cells with ugonin J treatment was directly mediated by p53, we generated p53-overexpressing MCF-7 cells. A 60% reduction of NANOG^+^ cells was found in p53-overexpressing MCF-7 cells, combined treatment with ugonin J further reduced 90% of NANOG^+^ cells. In contrast, treatment of pifithrin-*α* (p53 inhibitor) rescued the reduction of NANOG induced by ugonin J (Figures 6(b) and 6(c)). The results suggested that activation of p53 pathway mediated the effect of ugonin J on suppression of the NANOG expression.

### 3.5. Ugonin J Could Suppress Propagation of Breast Cancer Stem Cells *In Vivo*

**Figure 7:**
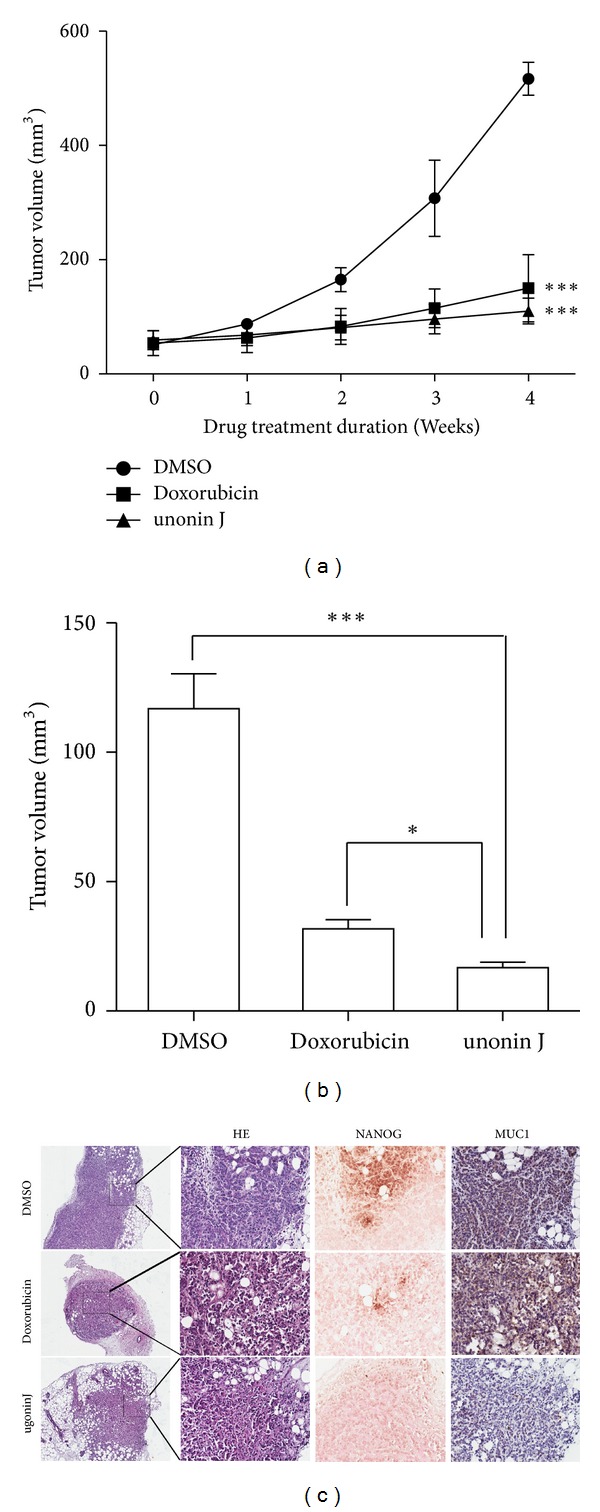
Effect of ugonin J on the growth of MCF-7 orthotopic tumor model. (a) SCID mice bearing MCF-7 orthotopic tumor were administrated weekly once with Doxorubicin (12 mg/kg) or ugonin J (50 mg/kg). Each group used 6 mice. The tumor volumes of SCID mice were measured weekly when the treatment began. (b) The average tumor volume of MCF-7 tumors was removed from SCID after 4 weeks. ***P < 0.001, **P < 0.01 versus DMSO control or Doxorubicin. (c) Hematoxylin-Eosin stain and immunohistochemical detection (×200) for NANOG and MUC1 on control, doxorubicin-treated, and ugonin J-treated tumor xenografts.

To further determine whether ugonin J can suppress the propagation of tumorigenic breast CSCs *in vivo, *MCF-7 cells (2 × 10^5^ cells) were injected into the mammary fat pads of female SCID mice. When the tumor volume reached 50 mm^3^ (Day 0), the tumor-bearing animals were administered 4 doses of doxorubicin (12 mg/kg) or ugonin J (50 mg/kg). As shown in Figures 7(a) and 7(b), ugonin J treatment significantly inhibited tumor propagation. Immunohistochemical analysis of tumor xengrafts further confirmed that treatment with ugonin J suppressed NANOG expression. In contrast, some Dox-treated cancer cells still expressed NANOG (Figure 7(c)) which can explain how tumors can still be slightly propagated (Figure 7(a)). These results suggest that ugonin J can suppress the propagation of breast CSCs *in vivo* via reduction of NANOG.

## 4. Discussion

NANOG is a transcriptional factor that plays key roles in the self-renewal and maintenance of pluripotency in embryonic stem cells [31]. There are 11 NANOG pseudogenes [32]. *NANOGP8* has been recognized as a retrogene and was recently found to be expressed in various cancer tissues and several cancer cell lines including the MCF-7 cells used in the current study. We have previously shown that activation of p53 by disrupting porphyrin homeostasis in embryonic stem cells resulted in suppression of NANOG expression [33]. In the current work, we observed a similar phenotype, where treatment of MCF-7 cells with cyclohexylmethyl flavonoids induced activation of p53, which in turn led to the reduction of NANOG expression. This suggests that NANOG expression is regulated by a similar mechanism in both breast CSCs and embryonic stem cells. Recent work further indicates that NANOG could be upregulated by beta-catenin through interaction with Oct3/4 [34]. We have evaluated the possibility by immunohistochemical analysis and Top/Fop flash assay (data not shown) and found that ugonin J treatment decreased the level of beta-catenin in tumor xengraft. However, the activity of beta-catenin was extremely low in both MCF-7 and MDA-MB231 cells. We therefore proposed that it is possible that Ugonin J treatment causes concomitant downregulation of beta-catenin and NANOG in breast cancer, but, in absence of wnt/beta-catenin, ugonin J is capable to downregulate NANOG expression through p53 activation. P53, a well-known tumor suppressor protein, involves regulating cell cycle, senescence, and apoptosis responses against the cell suffering from stress such as hypoxia or DNA damage. In most cancers, p53 is either lost or mutated to allow cancer cells to expand and progress [35]. Recent reports raised the possibility to suppress tumor growth by restoring wild-type p53 to cancer cells [36]. Our current work further highlights the importance of restoring the function of p53 in CSCs.

Recent work has further demonstrated that NANOG transcribed from the NANOGP8 locus is important in tumorigenesis [16]. RNA interference-mediated NANOG knockdown inhibited tumor development in xenograft animals and decreased long-term clonal and clonogenic growth of cancer cells [16, 17]. These results are consistent with our findings that overexpression of NANOG enhances the overall growth rate of MCF-7 cells and downregulation of NANOG by ugonin J treatment suppresses propagation of breast CSCs. However, the mechanisms, involved in regulating transcription of NANOG from the NANOGP8 locus during breast carcinogenesis, remain to be determined.

We have tried to determine the structure-activity relationship (SAR) of several cyclohexylmethyl flavonoids with high potency to suppress NANOG that may possess the specific structural features. We proposed that 6, 6-dimethyl-2-methylene-cyclohexylmethyl groups on the C-6 position are important for the potency of ugonins J and K to suppress propagation of breast CSCs. In contrast, the bulky isoprenyl group attached to position 2 of the B ring (found in ugonins M, N and O) may reduce the potency. In addition, the free rotation of the bulky isoprenyl moiety (ugonins J and K) may contribute more stereohindrance and lipophilic properties compared with the cyclized moiety with C-4 by ether-linkage (ugonins L and S), as the double bond in the cyclohexane (ugonin P) disrupts the chair form of the cyclohexane moiety and reduces its lipophilicity. And one hydroxyl group attached to the cyclohexyl ring (ugonins Q and R) might increase the hydrophilicity, which would also reduce the potency. It has been reported that derivatives of ambrein and agelasine that possessed cyclohexylmethyl groups are capable to suppress the expansion of multiple cancer cell lines [37, 38]. This may explain why ugonins J and K exhibited relatively high potency to suppress propagation of breast CSCs. 

CD24^-/low^CD44^+^ breast CSCs have been suggested to be the underlying cause of breast cancer recurrence and are a critical target for breast cancer therapies. *H. zeylanica* have been used in Chinese traditional medicine for treating inflammatory diseases and various hepatic disorders. In the present study, we have identified two cyclohexylmethyl flavonoids, ugonins J and K, which were the main components of the rhizomes of *H. zeylanica *and were able to suppress propagation of breast CSCs in mammosphere cultures and in tumor xengrafts. The current work also found that the suppressive effect of ugonin J on propagation of breast CSCs was mediated by activation of p53 which in turn led to reduction of NANOG. Overexpression of NANOG counteracted the suppressive effect of ugonin J. The current findings suggest that the rhizomes of *H. zeylanica* can possibly be used as complementary medicine for reducing CSC-mediated breast cancer recurrence.

## Figures and Tables

**Table 1:** Structures and activities of ugonins.

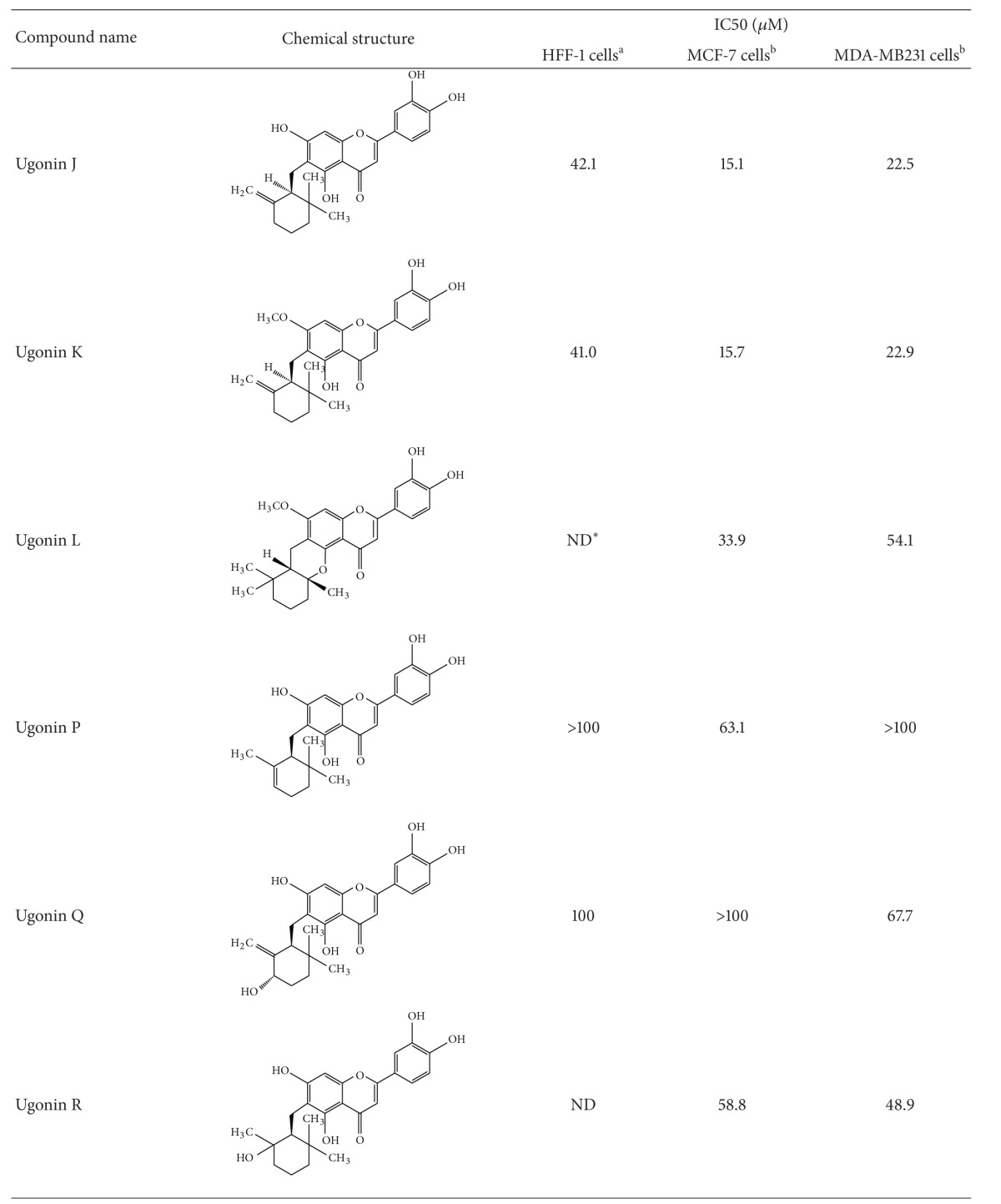 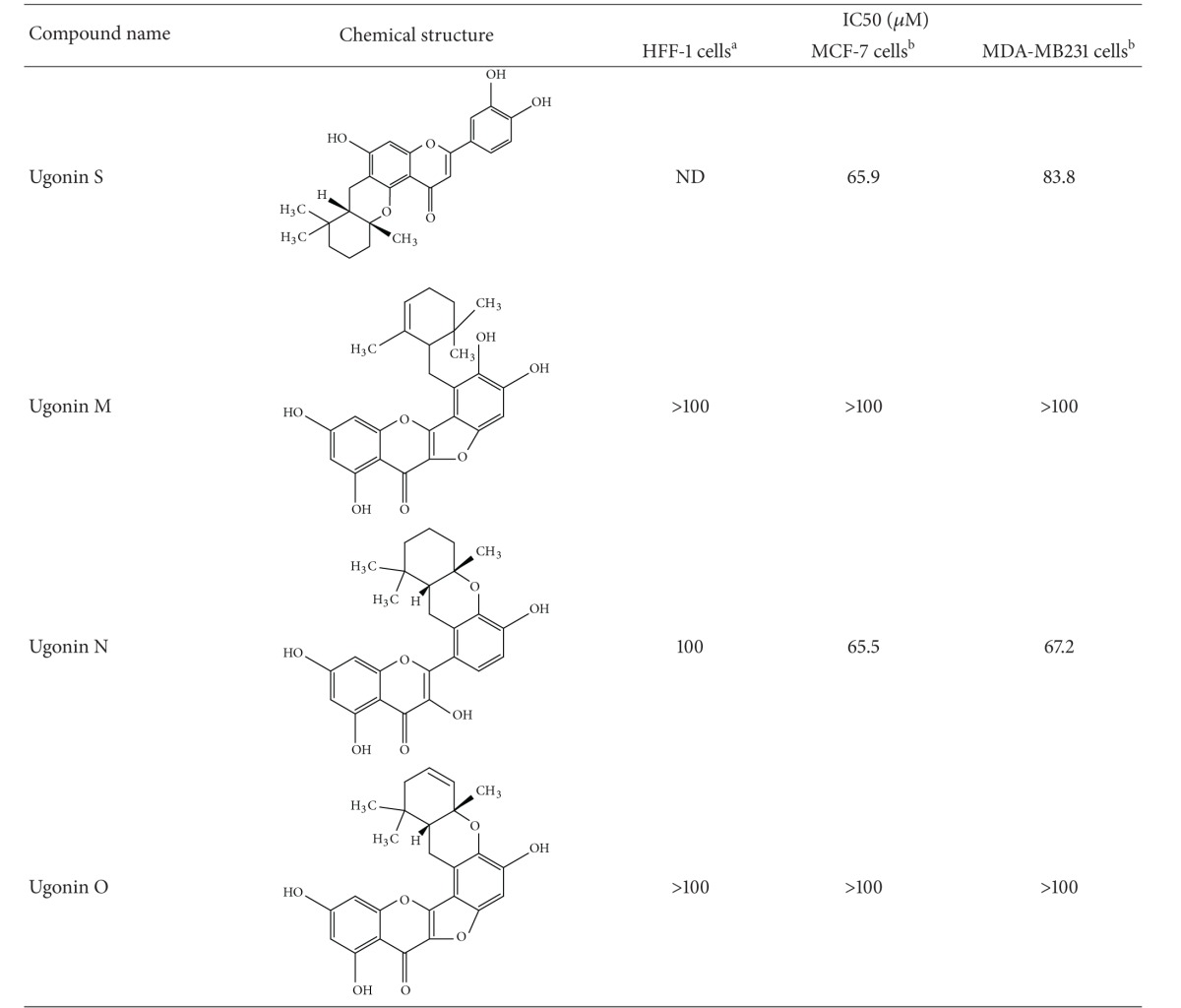

*ND: not determined. ^a^HFF-1: human foreskin fibroblasts and ^b^MCF-7/MDA-MB-231: human breast adenocarcinoma cell lines.
